# The complexities of maximising recruitment to complex intervention trials in child and adolescent mental health services

**DOI:** 10.1186/1745-6215-12-S1-A122

**Published:** 2011-12-13

**Authors:** David Cottrell, Liz Graham, Amanda Farrin

**Affiliations:** 1 School of Medicine, University of Leeds, Leeds, LS2 9NL, UK; 2 Clinical Trials Research Unit, University of Leeds, Leeds, LS2 9JT, UK

## Objectives

The HTA-funded SHIFT Trial (Self-Harm Intervention: Family Therapy) aims to recruit 832 young people following self-harm, along with their primary care givers. Recruitment is scheduled to take place over 3 years, across three ‘Hubs’ in the UK – Greater Manchester, London and Yorkshire – each involving approximately 15 Child & Adolescent Mental Health Services (CAMHS). Due to the complexities of set-up and the involvement of many clinicians in largely research-naïve services, recruitment has been slower than anticipated. The TMG thus agreed an intensive strategy to enhance the existing recruitment plan.

## Methods

The key approach to immediate improvement in recruitment rates was intensive work with existing services. The common approach of involving additional sites was not a straight-forward option, given that trial-specific Family Therapists (FTs) work across services and form a team for rotational delivery of FT in each service. Inclusion of new sites would involve either an increase in FT workload (which may or may not be possible should existing services increase recruitment), or development of an entirely new group of services involving lengthy and costly employment of additional Therapists, plus the governance approvals required for each Trust.

The main elements of this approach are a) passing on guidance regarding strategies employed in services already recruiting well to other CAMHS, b) a systematic, site-by-site approach with those identified as poor recruiters – this involves phone calls from the Hub Leads to each PI, increased Researcher contact, identification of SHIFT ‘Champions’ in each service location to ensure SHIFT is flagged at all relevant team meetings, c) MHRN CSO attendance at referral meetings to specifically flag eligible cases.

## Results

This approach was initiated at the end of February 2011. Sites have responded positively and have been keen to identify champions and look at strategies to ensure SHIFT is flagged as part of routine practice. To date (August 2011) we have seen a promising increase in recruitment.

## Conclusions

Strategies to ensure optimal recruitment in CAMHS require good local knowledge of the varying ways in which services operate to ensure a tailored approach to recruitment. Identification of Champions is proving successful. Increased Researcher and MHRN contact with sites, attendance at relevant team meetings, and maintenance of trial profile within each service is key.

